# Modelling invasive group A streptococcal disease using bioluminescence

**DOI:** 10.1186/s12866-018-1200-1

**Published:** 2018-06-19

**Authors:** L. E. Lamb, X. Zhi, F. Alam, M. Pyzio, C. L. Scudamore, S. Wiles, S. Sriskandan

**Affiliations:** 1 Section of Infectious Diseases and Immunity, Department of Medicine, Imperial College London, London, UK; 2 Royal Centre Defence Medicine, Academia and Research, University of Birmingham, Birmingham, B15 2SQ UK; 3 MRC Harwell, Harwell Science and Innovation Campus, Oxfordshire, OX11 0RD UK; 4 Bioluminescent Superbugs Lab, Department of Molecular Medicine and Pathology, Faculty of Medical and Health Sciences, University of Auckland, Auckland, New Zealand

**Keywords:** Bioluminescence, Biophotonic imaging, Group A Streptococcus, Infection model, Invasive disease, Luciferase, *Streptococcus pyogenes*

## Abstract

**Background:**

The development of vaccines and evaluation of novel treatment strategies for invasive group A streptococcal (iGAS) disease requires suitable models of human infection that can be monitored longitudinally and are preferably non-invasive. Bio-photonic imaging provides an opportunity to reduce use of animals in infection modelling and refine the information that can be obtained, however the range of bioluminescent GAS strains available is limited. In this study we set out to develop bioluminescent iGAS strains for use in in vivo pneumonia and soft tissue disease models.

**Results:**

Using clinical *emm*1, *emm*3, and *emm*89 GAS strains that were transformed with constructs carrying the *lux*ABCDE operon, growth and bioluminescence of transformed strains were characterised in vitro and in vivo.

*Emm*3 and *emm*89 strains expressed detectable bioluminescence when transformed with a replicating plasmid and light production correlated with viable bacterial counts in vitro, however plasmid instability precluded use in the absence of antimicrobial pressure. *Emm*89 GAS transformed with an integrating construct demonstrated stable bioluminescence that was maintained in the absence of antibiotics. Bioluminescence of the *emm*89 strain correlated with viable bacterial counts both in vitro and immediately following infection in vivo. Although bioluminescence conferred a detectable fitness burden to the *emm*89 strain during soft tissue infection in vivo, it did not prevent dissemination to distant tissues.

**Conclusion:**

Development of stably bioluminescent GAS for use in vitro and in vivo models of infection should facilitate development of novel therapeutics and vaccines while also increasing our understanding of infection progression and transmission routes.

**Electronic supplementary material:**

The online version of this article (10.1186/s12866-018-1200-1) contains supplementary material, which is available to authorized users.

## Background

Group A streptococcus (GAS) is responsible for a wide spectrum of diseases ranging from the mild, such as pharyngitis, to severe, including pneumonia and necrotizing fasciitis [1]. It is estimated that at least 663,000 cases of invasive GAS disease (iGAS) arise globally per year resulting in 163,000 deaths [2], with rates increasing in more recent years.

Mouse models are commonly used to enhance the understanding of iGAS pathogenesis and to assess different therapeutics and vaccines, although the scientific limitations of such models are well-recognised. Recently bio-photonic imaging (BPI) has been introduced to refine models and reduce the numbers of animals used in experiments, providing additional valuable information on the development of disease [3]. Models using bioluminescent bacteria must be carefully characterized, as bioluminescent bacteria can be attenuated following transformation with constructs containing the *lux* operon. BPI has been used to model nasopharyngeal GAS disease [4–6], although there has been limited use in invasive disease [7].

To refine animal models of iGAS using BPI, bioluminescent derivatives of dominant iGAS serotypes are required which produce enough light to be detected in vivo*,* demonstrate stability, and are not attenuated in comparison to the parent wildtype strains [4–6]. Although a commercially available *emm*49 bioluminescent GAS strain exists [8], the main *emm* types causing iGAS in the UK are *emm*1, *emm*3 and *emm*89 [9]. In this work we set out to develop strains to model iGAS using bioluminescent derivatives of these leading invasive *emm* types *emm*1, *emm*3, and *emm*89, employing a molecular strategy similar to that previously applied to *emm*75 GAS [6].

## Results

### Light expression, growth and stability of *emm*1, *emm*3 and *emm*89 GAS isolates transformed with a replicative plasmid containing the *lux* operon

**Fig. 1:**
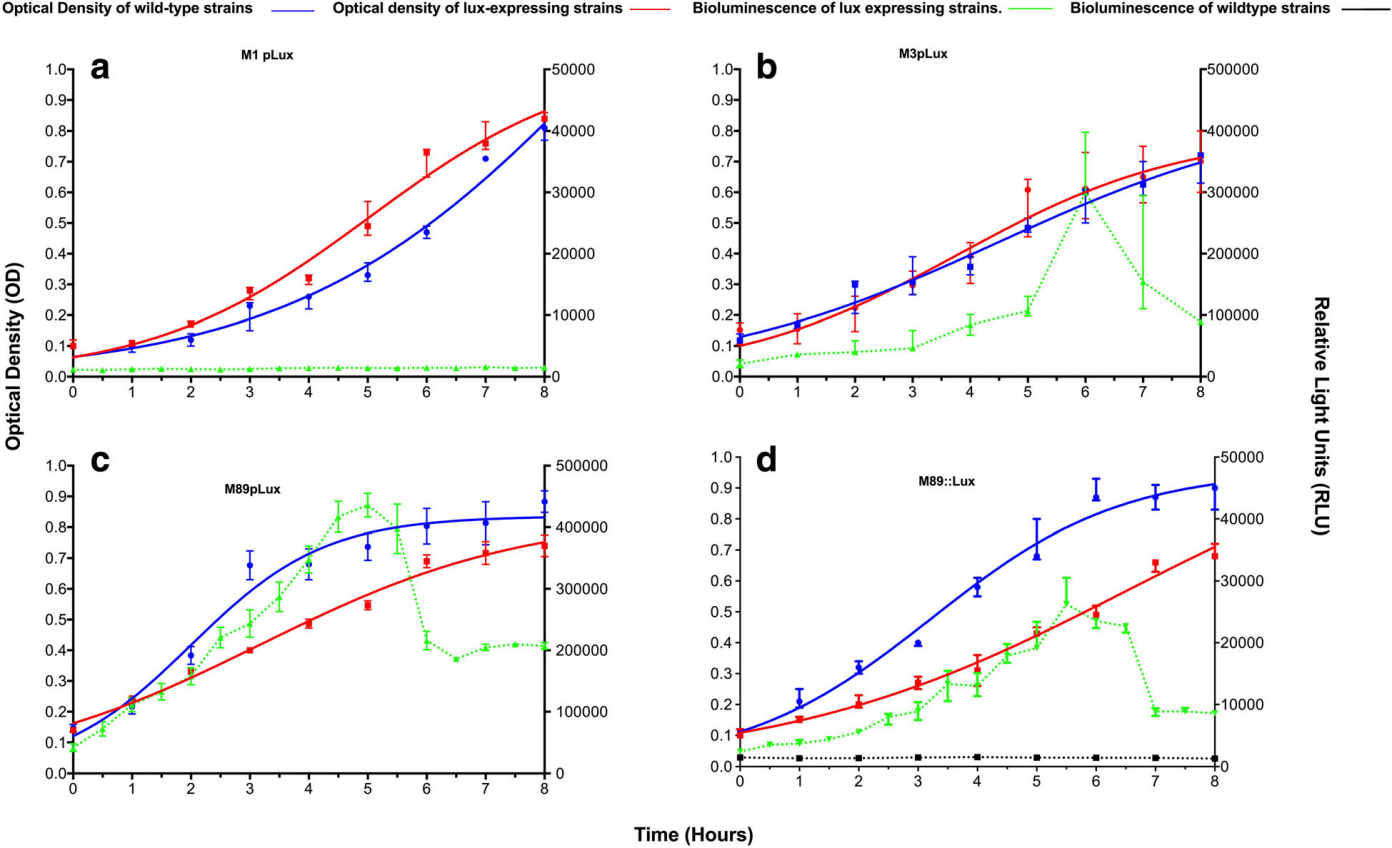
Growth kinetics and light production of M1 pLux, M3 pLux, M89 plux and M89::Lux. Growth of **a** M1pLux **b** M3pLux **c** M89pLux and **d** M89::lux (red lines) was compared with appropriate wild type parental strains (blue lines) over 8 h and a logistic curve was fitted to the growth data. Light was measured using a luminometer and recorded as relative light units (RLU) and is shown in green. Median and range of 3 biological replicate cultures are shown

Transformation of GAS with the *lux* operon on a replicative plasmid (pLux) resulted in light production by *emm*3 (M3pLux) and *emm*89 (M89pLux) strains, but not *emm*1 (M1pLux) (Fig. 1a-c). We observed that M1pLux maintained a functional *lux* operon despite not producing any light, as we were able to extract the plasmid, transform, and successfully express the operon in *Escherichia coli* (data not shown).

In contrast, M3pLux and M89pLux showed maximal light production after 5 and 6 h growth, respectively, with M3pLux peaking at approx. 3 × 10^5^ RLU and M89pLux peaking at approx. 4 × 10^5^ RLU (Fig. 1b, c). For both M3pLux and M89pLux, expression of light significantly correlated with optical density during in vitro growth over 8 h, although the relationship was strongest during exponential growth (Pearson’s *r* = 0.82) compared with the overall 8 h time period of culture (*r* = 0.63) for M3pLux and also for M89pLux (*r* = 0.96 during exponential growth compared with *r* = 0.49 overall). Expression of light attenuated in vitro growth by M89pLux but not M3pLux (*p* = 0.0003 and *p* = 0.4332, two-tailed paired *t*-test, respectively).

**Fig. 2:**
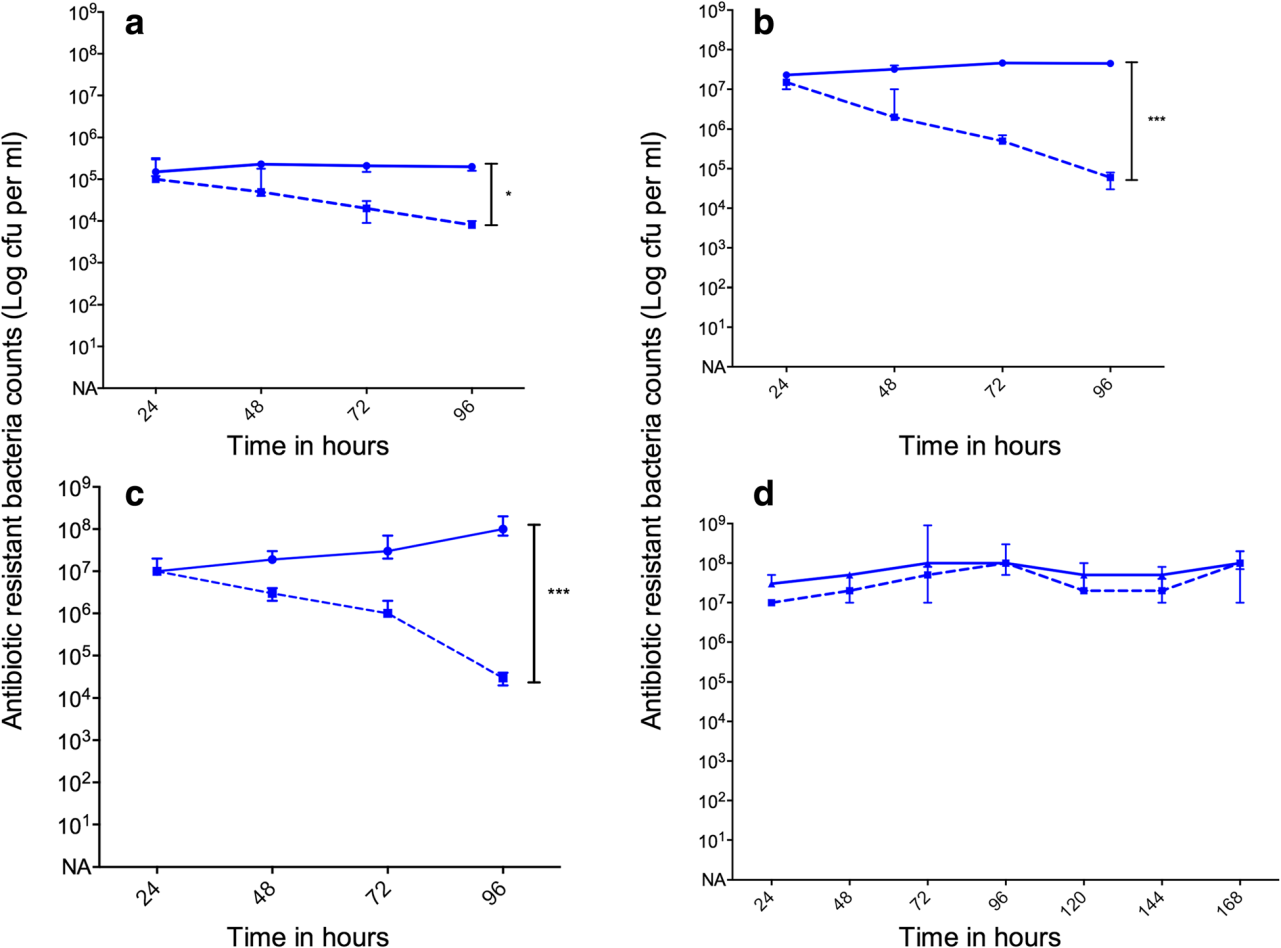
In vitro stability of transformed bioluminescent isolates: M1pLux, M3pLux, M89pLux and M89::Lux. Transformed strains **a** M1pLux **b** M3pLux **c** M89pLux **d** M89::Lux were passaged daily in THB with kanamycin (blue line) and without kanamycin (dashed blue line) to determine stability of the replicative plasmid pLux in vitro and the integrated plasmid pICL::lux. There was a significant reduction in the number of viable bacteria retaining the plasmid when passaged without kanamycin in comparison to the number of bacteria retaining the plasmid with kanamycin. Stability was determined by serial plating onto selective solid media. Median and range of three replicates per time point are shown

We observed differences in the in vitro stability of pLux in the absence of antibiotic selection (Two way ANOVA with Bonferroni Post-Test, Fig. 2a-c). Despite not conferring bioluminescence to *emm*1 GAS, plasmid pLux demonstrated instability in M1pLux GAS in the absence of antibiotic. There was no loss of plasmid by M3pLux within the first 48 h, although all plasmid was lost by 72 h. Plasmid pLux was stable in M89pLux for the first 24 h, but after this time the number of antibiotic-resistant cells declined slowly but steadily to a 4-log difference by the end of the experiment. Taken together, these results suggested that M3pLux and M89pLux might provide useful bioluminescence in vivo in the absence of selection for short (24-48 h) experiments.

### M3pLux and M89pLux GAS can be visualised non-invasively during lower respiratory tract infection, but are unstable

**Fig. 3:**
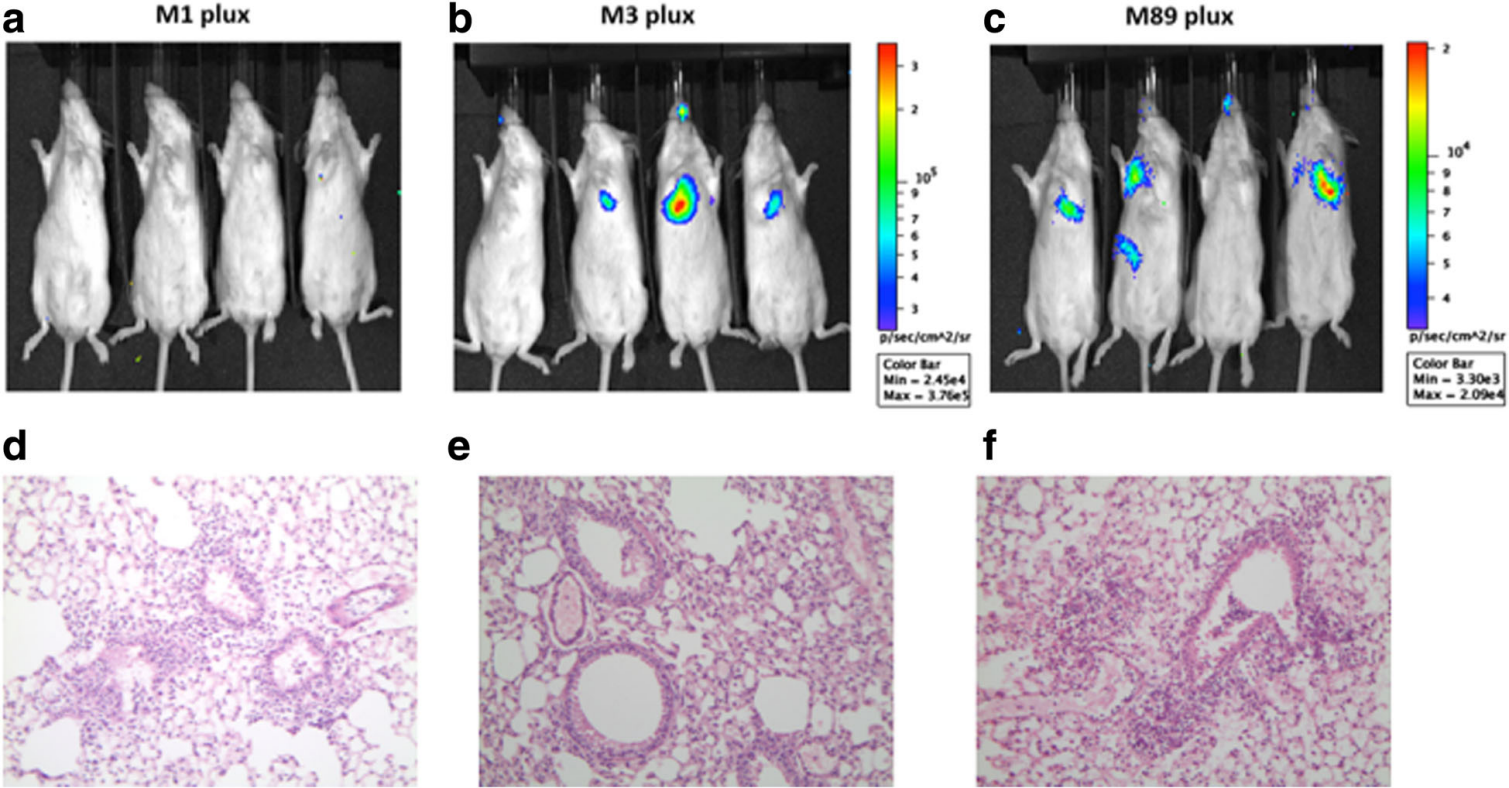
Evaluation of M1pLux, M3pLux and M89pLux in lower respiratory tract infection model. M1pLux, M3pLux and M89pLux were assessed in a mouse model of lower respiratory tract infection (LRTI) (*n* = 4 per group). IVIS images 2 h following intranasal administration of (**a**) M1pLux, (**b**) M3pLux and (**c**) M89pLux IVIS images were obtained. Ventral views of 4 individual mice imaged per group. Hematoxylin and eosin (**h** & **e**) stained sections of lung tissue following infection with M1pLux (**d**), M3pLux (**e**) and M89pLux (**f**) (× 20 magnification) showing inflammation with multifocal peribronchiolar infiltration by neutrophils and flooding of the alveoli by neutrophils, macrophages and proteinaceous fluid

Female CD1 mice age 6 to 8 weeks were infected intra-nasally with each plasmid-based bioluminescent strain to investigate ability to produce a lower respiratory tract infection (Fig. 3a-c). Mice were imaged at 2 and 4 h post infection, and experiments were stopped at 5 h post infection, as the mice reached defined sepsis end points. Visible light signals were seen from mice infected with M3pLux and M89pLux but not M1pLux, consistent with the in vitro findings. In one mouse infected with M89pLux, bacteria were seen by bioluminescence to have disseminated from the lungs (Fig. 3c), and bacterial counts were subsequently recovered from the liver (data not shown). All three GAS strains, M1pLux, M3pLux, and M89pLux resulted in a productive infection of the lower respiratory tract; lungs of infected mice demonstrated an inflammatory response 2 h following infection, with infiltration of the alveoli with neutrophils, macrophages and proteinaceous fluid (Fig. 3d-f).

Importantly, however, despite the observed 24-48 h in vitro stability of pLux in the absence of antibiotic, only 60–70% and 50–70% of the M3pLux GAS colonies recovered from lung homogenates retained the pLux plasmid after 2 and 4 h, respectively. The plasmid was even less stable in M89pLux, and was present in only 17–23% and 0.5–27% of the M89pLux GAS colonies recovered from lung homogenates after 2 and 4 h, respectively.

### Development and in vitro characterization of stable bioluminescent M89 GAS

To circumvent the problems inherent in plasmid loss, chromosomal integration of the *lux* operon was employed as an alternate strategy. Targeted integration of the pICL18^Lux^ plasmid into gene *spy0535* was confirmed in M89::Lux using PCR as previously described [5]. Primers LF and RR amplified a product of 10 kB in M89::Lux and a smaller product of 839 bp equivalent to the size of *spy0535* in the parent *emm*89 GAS strain (Additional file 1: Figure S1).

We observed that M89::lux exhibited maximal light production (approx. 3 × 10^4^ RLU) at 5.5 h (Fig. 1d), but light levels were 10-fold lower than the same strain expressing the *lux* operon from plasmid pLux. Expression of light significantly correlated with optical density during in vitro growth over 8 h, although the relationship was stronger during exponential growth (Pearson’s *r* = 0.45 [*p* = 0.0189] over 8 h and *r* = 0.98 [p = < 0.0001] during exponential growth). Similar to transformation with pLux, chromosomal expression of the *lux* operon significantly attenuated in vitro growth (p = < 0.0001, two-tailed paired *t*-test). In contrast to *emm*89 GAS transformed with pLux, there was no loss of the *lux* operon by M89::Lux after in vitro growth without antibiotic selection for 168 h (Fig. 2d). Targeted disruption of the Spy0535 locus using plasmid lacking the *lux* operon had limited effect on growth in contrast to pICL18^Lux^ (Additional file 2: Figure S2).

### Use of stably bioluminescent M89::Lux GAS strain in lower respiratory tract infection

M89::lux was administered to mice intra-nasally to produce lower respiratory tract infection. Despite using the same bacterial inoculum as used in earlier studies, a bioluminescent signal was not observed in vivo. This was despite the fact that the infection led to histopathological evidence of lung inflammation (Additional file 3: Figure S3A) and that all M89::Lux GAS retained antibiotic resistance when tested (not shown). However, following a direct intrathoracic infection post mortem using the same inoculum dose, it was possible to detect a bioluminescent signal in lungs that were dissected (Total Flux 182,400 photons/second) thus we concluded that the signal was insufficiently strong to traverse the thoracic cage, despite the burden of bacterial infection at that time point being 2 × 10^8^ cfu/lung (Additional file 3: Figure S3B).

### Use of stably bioluminescent *emm*89 GAS strain in soft tissue infection

**Fig. 4:**
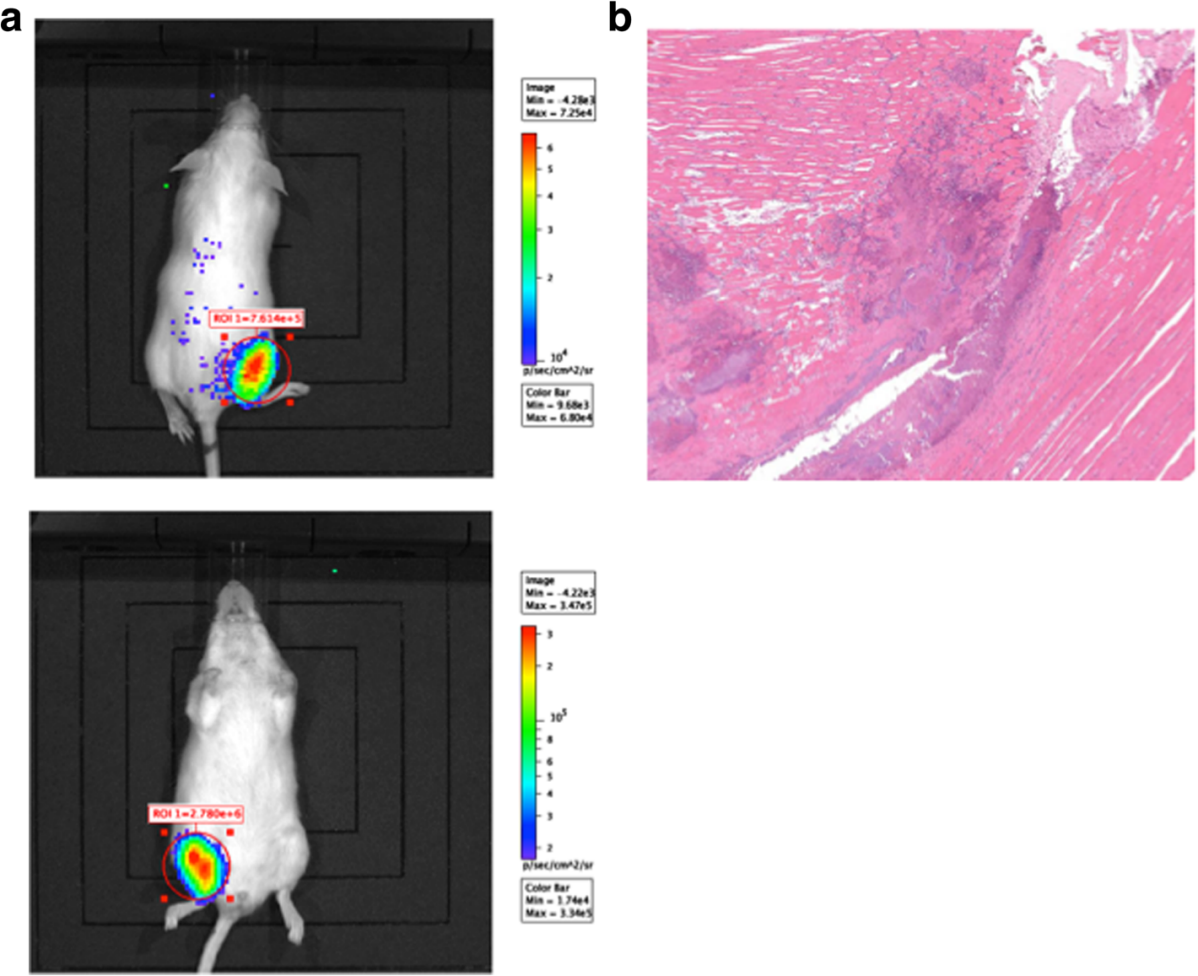
Evaluation of M89::Lux following intramuscular infection**. a** Bioluminescent signal from a single mouse representative of six mice tested showing ventral (lower panel) and dorsal (upper panel) viewpoints immediately following an i.m. infection with the bioluminescent strain M89::Lux. **b** Haematoxylin and eosin stained section of muscle tissue 24 h following an intramuscular infection with M89::Lux demonstrating abundant bacterial growth, myonecrosis and localised inflammatory infiltrate. Magnification × 20

Having established that a lower respiratory tract infection may be difficult to monitor non-invasively, M89::Lux was then assessed in a soft tissue model of invasive disease. Following intramuscular infection with M89::Lux a bioluminescent signal was immediately observed in the thigh of infected animals (Fig. 4a) that demonstrated significant correlation (r^2^ = 0.88, *p* < 0.05 logistic regression) with the actual bacterial burden quantified from the tissue (Additional file 4: Figure S4). Although light was also observed 24 h following infection, the bioluminescent signal no longer correlated with bacterial burden (data not shown), despite there being a detectable inflammatory infection in the thigh, with histopathology showing extensive areas of necrosis affecting muscle and surrounding tissue with inflammation and bacterial colonies (Fig. 4b).

### Fitness burden of bioluminescence expression by *emm*89 GAS

In previous work [5] we determined that expression of light by GAS derivative M75::Lux conferred an overall fitness burden that was manifest in vivo as a reduced growth rate and reduced competitive index when compared with the parent strain. Accordingly, mice were infected intramuscularly with experimental inocula containing a 50:50 mix of M89::Lux and M89 wild type GAS. After 24 h, mice were euthanised and the ratio of M89::Lux to parent *emm*89 strain was calculated (by replica plating of colonies onto selective media) from both the site of infection, and also sites of dissemination including the local draining lymph node, blood, spleen, and liver (Table 1). Expression of *lux* by M89::Lux conferred a measurable fitness burden that could be detected at most sites of infection; indeed there was limited systemic dissemination by the bioluminescent strain to the liver and blood (Table 1). The fitness burden of the *lux* operon in both M89::Lux and M75::Lux GAS was also observed using larvae of the Greater wax moth *Galleria mellonella* as a surrogate host although did not reach significance (Additional file 5: Figure S5).

**Table 1:** Fitness burden conferred by bioluminescence in M89::Lux during soft tissue infection

	Thigh muscle	Lymph node	Spleen	Liver	Blood
Number of mice with bacteria (WT or BLI strain) present^a^	6/6	3/6	4/6	3/6	3/6
Median Competitive index^b^ (range)	0.70 (0.66–1.16)	1.9 (0.8–3)	0.25 ^c^	^d^	^d^

^a^Mice infected i.m. with equal inocula of M89 and M89::Lux. Samples taken 24 h after infection. Number of mice where any GAS bacteria could be cultured is indicated (wildtype or bioluminescent strain)

^b^Competitive index calculated as M89::Lux cfu/M89 cfu and could only be calculated for mice with detectable bacteria. A value of 1 indicates no fitness burden. M89::Lux was distinguished from the parent strain by replica plating onto selective media

^c^ No range because only one CI could be calculated

^d^Competitive index could not be calculated because only WT disseminated to blood or liver (i.e. CI = 0)

### Comparison of bioluminescent M89::Lux and M75::Lux in invasive soft tissue infection and systemic spread

The ability of different bioluminescent strains to be used in invasive models of GAS infection was assessed by comparing dissemination of M89::Lux to the ipsilateral lymph node, blood, spleen and liver, with the previously characterised M75::lux pharyngitis strain [5, 6]. Female CD1 mice aged 6–8 weeks were infected intramuscularly with the bioluminescent strains M89::Lux and M75::Lux. At 24 h following infection mice were euthanised and the following organs harvested: thigh, ipsilateral inguinal lymph node, spleen, liver and blood to assess the bacterial burden in the tissues. Bioluminescence of colonies cultured on solid media from the tissues was analysed, to assess the stability of the Lux chromosomal construct in the strains. M89::Lux demonstrated increased stability at the site of infection and at sites of dissemination in comparison to the M75::Lux strain. Interestingly, in the absence of competition from the parent strains, both the bioluminescent *emm*89 and *emm*75 strains disseminated systemically. The M89::Lux strain remained genetically stable on dissemination to tissues, in contrast to M75::Lux which, despite stability at the site of infection, demonstrated loss of the construct on dissemination (Table 2).

**Table 2:** Stability of Lux construct function during intramuscular infection with M89:Lux or M75::Lux

	Thigh muscle	Lymph node	Spleen	Liver	Blood
M89::lux					
Number of mice with bacteria present after 24h^a^	6/6	1/6	2/6	3/6	0/6
% colonies bioluminescent ^b^(Number counted)	89% (90/110, 293/300, 55/70, 49/50, 40/60, 40/65)	100% (10/10)	100% (50/50, 50/50)	99% (43/44, 10/10, 50/50)	N/A
M75::Lux					
Number of mice with bacteria present after 24h^a^	6/6	3/6	2/6	2/6	4/6
% colonies bioluminescent ^b^ (Number counted)	75% (40/80, 50/85, 39/55, 55/80, 40/60, 45/50)	0% (0/5, 0/37, 0/50)	0% (0/10, 0/30)	0% (0/20, 0/20)	0% (0/10, 0/10, 0/10, 0/20)

^a^Mice infected i.m. with either M89::Lux or M75::Lux. Samples taken 24 h after infection. Number of mice where bacteria could be cultured is indicated

^b^Stability of the Lux construct was determined by determining % colonies that demonstrated bioluminescence. Where possible at least 100 colonies were counted for each tissue and each mouse

## Discussion

Bioluminescence imaging has refined animal models, allowing infections to be monitored non-invasively and in real-time, reducing the number of animals required to carry out experiments and providing further information regarding the development of disease [9]. In this work, we have developed bioluminescent derivatives of invasive GAS and assessed these in different models of infection.

The bioluminescent strains developed in this work demonstrated light production that correlated with bacterial quantities in vitro, a central requirement for use of BPI in modelling infection. Although the multi copy plasmid constructs used in our work conferred greater brightness to *emm*3 and *emm*89 GAS, these derivatives were suitable only for very short-term studies due to poor stability. *Emm*1 GAS failed to produce light in vitro or in vivo despite confirmation that the bacterial *lux* operon construct was functionally intact, suggesting that the environment created by *emm*1 GAS might not support expression of the *lux* operon for reasons that are not fully understood at present. Others have also reported reduced light production in *emm*1 GAS using the *lux* operon although constructs transformed using the firefly luciferase (FFluc) can produce light in *emm*1 GAS [10]. FFLuc constructs require exogenous administration of a substrate, luciferin, for light production whereas the *lux* operon encodes the genes for the luciferase enzyme and recycling of the aldehyde substrate. As such, it could be that substrate expression or availability is reduced in *lux*-transformed *emm*1 GAS. Although use of FFLuc may circumvent this, the need for an exogenous substrate is more challenging in vivo if an injectable agent such as luciferin is required, since our own imaging studies have shown that mice infected via the respiratory tract can auto- and cross-infect such injection sites readily, simultaneously highlighting the superiority of BPI to demonstrate hitherto unrecognised transmission routes for infection.

Despite the brightness of bioluminescent strains containing the replicative plasmid, pLux, instability during passage made these strains undesirable for use in vivo. Plasmid instability can be overcome using toxin-antitoxin (T-AT) strategies that prevent loss of daughter cells that do not contain the target plasmid [11]. To circumvent problems of plasmid instability in the current work, a more stably bioluminescent *emm*89 strain was developed through chromosomal integration of the *lux* operon. As expected from previous studies [5] the strain was tenfold less bright than its counterpart strain transformed with a replicative plasmid, and showed attenuation in growth in broth compared with the parent strain and a control strain with disruption of Spy0535 alone. Previous work to create M75::Lux demonstrated that disruption of the chromosomal Spy0535 locus had no impact on growth or fitness of GAS in vitro or in vivo*;* although a clear fitness burden was directly related to insertion and expression of the *lux* operon [5]. We speculate that a similar fitness burden is conferred by the *lux* operon in M89::Lux although the degree to which this affects use in animal models of infection is not yet clear*.*

M89::Lux was not bright enough to be non-invasively detected in vivo during the early stages of inflammation and was only visualised after exposure of the lungs by dissection. Although there was a significant correlation between bacterial burden and light emitted from the dissected lung, similar to that reported by other groups [11], this mode of measurement was not practical and was only found following a direct intrathoracic injection. We cannot exclude the possibility that imaging of lung inflammation might have been more successful at later time points, or if the bacterial inoculum had been prepared using bacteria at logarithmic growth, given that bioluminescence expression is greatest during late logarithmic growth.

In contrast, M89::Lux was successfully used to model infection in the thigh, and light production in vivo correlated with bacterial counts in the early stages of infection, a feature that is under-reported in the literature. Importantly however, there was a reduction in correlation between bacterial burden and light emitted after 24 h. The possible reasons for this are manifold but include conditions that limit the activity of the *lux* operon such as anaerobiasis in tissues, or reduced bacterial metabolic activity as a starvation response during GAS deep soft tissue infection. Perhaps unsurprisingly, the expression of the *lux* operon by M89::Lux conferred a measurable fitness burden in vivo that could be detected at all sites of infection; indeed there was limited systemic dissemination by the bioluminescent strain in comparison to the parent strain. The extent to which such a fitness burden matters largely depends on the model or characteristic evaluated. It seems likely that subtle genetic differences in virulence between isogenic bioluminescent strains may be obscured by such a fitness burden while larger effects such as those conferred by vaccination or antimicrobial therapy could be detected easily.

The M89::Lux and M75::Lux bioluminescent strains were compared in the invasive soft tissue infection model of iGAS. Despite chromosomal integration of the *lux*ABCDE construct, and stability of the M75::Lux isolate in the nasopharynx, it was apparent that some loss of the construct occurred upon systemic dissemination of GAS to distant systemic sites, which was only apparent for M75::Lux and not the newly constructed M89::Lux strain. While this no doubt relates to the use of an otherwise intact plasmid to achieve chromosomal integration, as well as increased host immune pressure during bacterial systemic spread, the stability of M89::lux underlines potential differences between strains depending on the model used and provides a bioluminescent strain that can be more readily used in soft tissue infection models.

## Conclusions

We used bioluminescent GAS in in vitro and in vivo models of infection to facilitate the evaluation of novel therapeutics and vaccines while also increasing our understanding of infection progression and transmission routes. Despite some attenuation, the newly derived *emm*89 bioluminescent GAS represents a clinically invasive strain suitable for use in soft tissue infection studies.

## Methods

### Bacterial strains

The GAS strains used in this study are shown in Table 3. Clinical strains H305 (*emm*1) and H293 (*emm*89) have been extensively characterised in previous laboratory studies [13, 14] while strain H325, that was isolated from blood as part of routine clinical care, was selected as the most transformable clinical *emm*3 strain available. GAS strains were cultured in Todd Hewitt Yeast broth (THY), Todd Hewitt Agar (THA) or on Columbia Blood Agar (CBA). Plasmids were propagated in *Escherichia coli* strain DH5α TOP10 Competent Cells (Invitrogen) and grown in Luria Bertani (LB) broth at 37 °C shaking at 200-220 rpm. For stability experiments, bioluminescent strains were passaged daily in the presence and absence of antibiotics and then replica plated onto CBA and antibiotic selective media. For *E. coli*, ampicillin (Sigma-Aldrich, UK) was used at 50 μg/ml, while for GAS, kanamycin (Sigma-Aldrich, UK) was used at 400 μg/ml. All strains were grown at 37°C.

**Table 3:** GAS strains used in this study

M type	Strain ID	Source	Reference
M1	H305	Scarlet fever (NCTC8198) and necrotising fasciitis models	[13]
M3	H325	Necrotising fasciitis/blood	This study
M75 M75::Lux	H347 H347::Lux	Tonsillitis	[5, 6]
M89	H293	Necrotising fasciitis	[14]

For animal experiments, GAS were cultured without shaking with 5% CO_2_ at 37C overnight and centrifuged (Sorvall RTH 750 Rotor), washed twice in phosphate buffered saline (PBS), and re-suspended in PBS to produce an inoculum of 1 × 10^7^ – 1 × 10^8^ colony forming units (cfu) per mouse. Numbers of viable bacteria within the different inocula were retrospectively assessed through plating serial dilutions of each inoculum onto blood and antibiotic selective agar. Bioluminescence was measured in vitro at regular time intervals as relative light units (RLU) using a bench top luminometer (Modulus).

### Plasmids and construction of bioluminescent derivatives

Two plasmids were used to introduce l*ux*ABCDE into *emm*1, *emm*3, and *emm*89 strains by electroporation as described previously; [12] pLux (pTHLK) [5] which is a replicative plasmid and pICL18^Lux^ which acts as a suicide vector following introduction of *lux*ABCDE into the redundant Spy0535 locus by homologous recombination. Disruption of the Spy0535 locus in *emm*89 was achieved as previously [5] using plasmid pUCMUTΔ0535 [14].

### Animal experiments

6-week-old female CD1 (Harlan, UK) were used to model infections. Mice were maintained in HEPA filtered cages with sterile bedding and free access to sterilized food and water. The number of mice per cage was dependent on weight. GLP Mini Fun Tunnels (Lillico) were provided in the cages for environmental enrichment. In vivo experiments were performed in accordance with the Animals (Scientific Procedures) Act 1986, subject to protocols that were approved by the Imperial College Ethical Review Process (ERP) panel and the UK Home Office. At the end of experiments, all the animals were culled under Schedule 1 to the Animals (Scientific Procedures) Act 1986 using CO_2_ inhalation and then confirmation of death by dislocation of the neck.

### Murine model of lower respiratory tract infection

Following gaseous anaesthesia with isofluorane, CD1 mice were challenged intranasally with 1 × 10^7^ - 1 × 10^8^ cfu per mouse, administered in a volume of 25 μl. Pilot work has shown this volume and bacterial load to be sufficient to cause a reproducible lower respiratory tract infection and lung inflammation. Viable bacteria within inocula were assessed by serial dilutions onto the appropriate media. Animals were imaged at 0 h, 2 h, and 4 h after infection and all experiments terminated when animals reached planned endpoints. Bacterial burden in the nose, lung, spleen, liver, blood, muscle and inguinal lymph node (ILN) was quantified. To determine whether the chest wall attenuated the bioluminescent signal obtained from the strains, a known inoculum of M89::Lux was administered directly into the thoracic cage of CD1 mice immediately after being euthanised.

### Murine intramuscular infection

Female CD1 aged 6 to 8 weeks were intramuscularly infected with approximately 1 × 10^8^ cfu in a 50 μl volume by injection directly into the thigh [13]. Following infection the animal was allowed to recover and move freely in the cage. Viable bacteria within the inoculum were quantified by plating serial dilutions. Animals were observed for 24 h following infection, euthanised at the defined end point and infected muscle, inguinal lymph nodes, spleen, liver and blood were cultured to quantify GAS. To determine stability of constructs in vivo, bacterial colonies from dissected tissues were plated onto TH agar and bioluminescence quantified after 5 h of growth (Modulus Single Tube Luminometer). The proportion of colonies of *S. pyogenes* that were bioluminescent (or kanamycin resistant) was quantified to determine the proportion of each strain surviving in vivo.

#### In vivo assays of fitness burden

For competition assays, GAS strains were cultured overnight, centrifuged (1864×g for 10 min) and washed twice in PBS. The optical density of each strain at 600 nm (OD600) for each strain was adjusted to 1 (7 × 10^9^ CFU ml^− 1^), and suspensions of different isolates were mixed 1:1 pairwise and used to inoculate mice intramuscularly. Bacterial colonies from dissected tissues were replica plated onto selective media. Individual colonies were inoculated into TH agar and bioluminescence quantified after 5 h of growth (Modulus Single Tube Luminometer). GAS that were either bioluminescent or kanamycin resistant were quantified to determine the proportion of each strain surviving in vivo. Competitive Indices (CI) were calculated as follows: CI = (mutant output/WT output)/(mutant input/WT input).

#### Galleria mellonella fitness assay

To determine fitness of GAS strains in an alternate surrogate host with a functioning innate immune system, *Galleria mellonella* were obtained from a single vendor (Live foods) and infected with 10 μl GAS or PBS (control) into the left hindmost proleg, as per previously described [15, 16]. Following inoculation, the infected larvae were incubated at 37 °C in 5% CO_2_ in petri dishes. The wax worm larvae were infected in groups of 20 and observed for survival with mortality of the wax worm shown by rapid melanisation (dark pigmentation) and no movement.

### Imaging

Groups of infected mice were subjected to bioluminescence analysis under isofluorane anaesthetic using an IVIS 100 (Perkin Elmer, MRC Biological Imaging Centre) or IVIS Lumina (Perkin Elmer, kind access Lung Immunology Group). During imaging, a reference image was obtained of the animal under low illumination prior to quantification of photons emitted from bioluminescent GAS strains at a binning of 4 over 60 s. Photon emission was determined using the Living image Software package (Version 3.2) and region of interests (ROI) were calculated and displayed as total flux and then compared to bacterial counts in the same organ tissue.

### Histopathology

Samples of tissue were formalin-fixed, decalcified, paraffin-embedded, sectioned and stained using haematoxylin and eosin (H&E) and Gram’s stain. Sections included lung and muscle and were examined by an experienced pathologist (CS).

### Statistics

For statistical analysis of growth curves, the Spearman rank correlation test was applied. For statistical analysis of colony count comparisons, a non-parametric Kruskal-Wallis test and Dunn’s post-test were used. For the in vitro work comparing growth of the strains a non-linear regression (logistic regression) test was used. Further comparisons were made using the two-way ANOVA with Bonferroni post-test and the *t*-test. *P* values less than 0.05 were defined as significant. Statistical analyses were performed using Graph pad Prism (version 6). Data are presented as median with an interquartile range.

## Additional files

Additional file 1: **Figure S1**. Construction of M89::Lux. The integrating plasmid pICL18^Lux^ is shown with the target of integration spy0535 highlighted. A representation of the integration of the plasmid into the M89 genome via a single crossover is shown and the position of the diagnostic primers LF and RR. (PNG 50 kb)

Additional file 2: **Figure S2**. Disruption of Spy0535 with plasmid control. Growth of M89 (blue), M89Δ0535 (purple, with disruption of Spy0535 using pUCMUTΔ0535) and M89::lux (red) was compared over 8 h. Mean and standard deviation of 3 biological replicate cultures are shown. (PNG 160 kb)

Additional file 3: **Figure S3**. Use of M89::Lux to model LRTI. A. Histopathology of lung tissue illustrating the inflammatory response 24 h following intranasal infection with M89::Lux. **B.** Light emitted from dissected mouse lung following direct intrathoracic injection of M89::Lux at 0 h; no light was visible through body wall. **C** Relation between light (total flux) and bacterial load (cfu) in dissected lung, *n* = 5. (PNG 307 kb)

Additional file 4: **Figure S4**. Bacterial load and light emission following intramuscular infection with M89::Lux. A. Correlation immediately after infection between bacterial load in thigh muscle and total flux obtained dorsally using six mice, r^2^ = 0.88, (*p* < 0.05). B Lack of significant correlation between bacterial burden and total flux obtained ventrally (r^2^ = 0.77). (PNG 113 kb)

Additional file 5: **Figure S5**. Fitness burden conferred by bioluminescence in *Galleria* infection. Virulence of parent strains, M89 (blue line) and M75 (red line) was compared with bioluminescent derivatives M89::Lux (dotted blue line) and M75::Lux (dotted red line) in *Galleria mellonella* survival assays (*n* = 20 larvae per group). Larvae were inoculated with 1-2 × 10 ^6^ cfu and monitored daily for survival over 5 days. Kaplan-Meier survival plots of infected larvae are shown compared with a PBS-inoculated group (controls – black line). Differences between strains were not significant. (PNG 38 kb)

## Abbreviations

BPI: Biophotonic Imaging
GAS: Group A Streptococcus

## References

[1] Stevens DL (1995). Streptococcal toxic-shock syndrome: spectrum of disease, pathogenesis, and new concepts in treatment. Emerg Infect Dis.

[2] Carapetis JR, et al. The global burden of group **A** streptococcal diseases. Lancet Infect Dis. 2005;5(11):685–94. 10.1016/S1473-3099(05)70267-X 16253886

[3] Sims Sanyahumbi A, et al. Global Disease Burden of Group A Streptococcus. 2016 In: Ferretti JJ, Stevens DL, Fischetti VA, editors. Streptococcus pyogenes : Basic Biology to Clinical Manifestations. Oklahoma City: University of Oklahoma Health Sciences Center; 2016-. Available from: https://www.ncbi.nlm.nih.gov/books/NBK333415/. 26866218

[4] Andreu N (2010). Optimisation of bioluminescent reporters for use with mycobacteria. PLoS One.

[5] Alam FM (2013). Non-invasive monitoring of Streptococcus pyogenes vaccine efficacy using biophotonic imaging. PLoS One.

[6] Alam FM (2013). Inactivation of the CovR/S virulence regulator impairs infection in an improved murine model of Streptococcus pyogenes naso-pharyngeal infection. PLoS One.

[7] Seok J (2013). Genomic responses in mouse models poorly mimic human inflammatory diseases. Proc Natl Acad Sci U S A.

[8] Takao K, Miyakawa T (2015). Genomic responses in mouse models greatly mimic human inflammatory diseases. Proc Natl Acad Sci U S A.

[9] Andreu N, Zelmer A, Wiles S (2011). Noninvasive biophotonic imaging for studies of infectious disease. FEMS Microbiol Rev.

[10] Cleary PP, et al. Immunization with C5a peptidase from either group a or B streptococci enhances clearance of group **A** streptococci from intranasally infected mice. Vaccine. 2004;22(31–32):4332–41. 10.1016/j.vaccine.2004.04.030 15474726

[11] Sheel M, et al. Correlation between bioluminescence and bacterial burden in passively protected mice challenged with a recombinant bioluminescent M49 group **A** streptococcus strain. Clin Vaccine Immunol. 2010;17(1):127–33. 10.1128/CVI.00256-09 PMC2812104 19889937

[12] Turner CE (2009). Emerging role of the interleukin-8 cleaving enzyme SpyCEP in clinical Streptococcus pyogenes infection. J Infect Dis.

[13] Sriskandan S, et al. Molecular analysis of the role of streptococcal pyrogenic exotoxin **A** (SPEA) in invasive soft-tissue infection resulting from Streptococcus pyogenes. Mol Microbiol. 1999;33(4):778–90. 10.1046/j.1365-2958.1999.01525.x 10447887

[14] Sriskandan S, et al. Mitogenic factor (MF) is the major DNase of serotype M89 Streptococcus pyogenes. Microbiology. 2000;146(Pt 11):2785–92. 10.1099/00221287-146-11-2785 11065357

[15] Olsen RJ, et al. Virulence of serotype M3 group **A **Streptococcus strains in wax worms (**G**alleria mellonella larvae). Virulence. 2011;2(2):111–9. 10.4161/viru.2.2.14338 PMC3100763 21258213

[16] Loh JM, et al. Galleria mellonella larvae as an infection model for group **A** streptococcus. Virulence. 2013;4(5):419–28. 10.4161/viru.24930 PMC3714134 23652836

