# Supplementary figures and images for: Modelling invasive group A streptococcal disease using bioluminescence
Source: BMC Microbiol. 2018 Jun 19;18:60. doi: 10.1186/s12866-018-1200-1 (PMC6006931; doi:10.1186/s12866-018-1200-1)

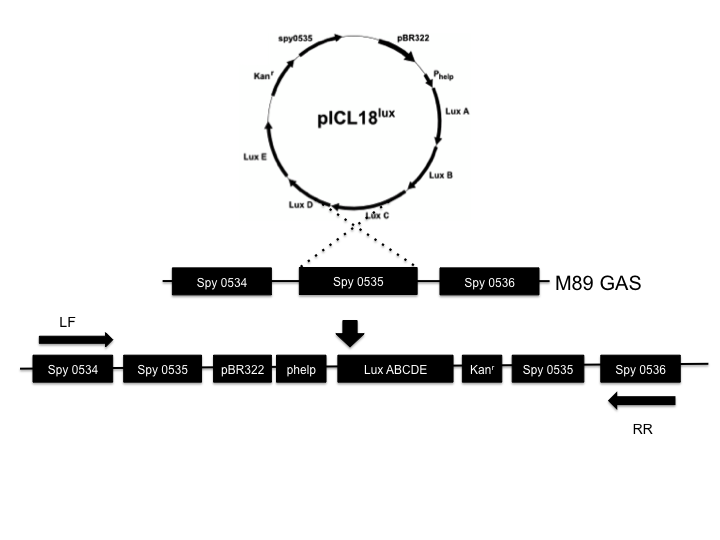

Supplement: Supplementary file 1 — Figure S1. Construction of M89::Lux. The integrating plasmid pICL18Lux is shown with the target of integration spy0535 highlighted. A representation of the integration of the plasmid into the M89 genome via a single crossover is shown and the position of the diagnostic primers LF and RR. (PNG 50 kb) [file 12866_2018_1200_MOESM1_ESM.png]

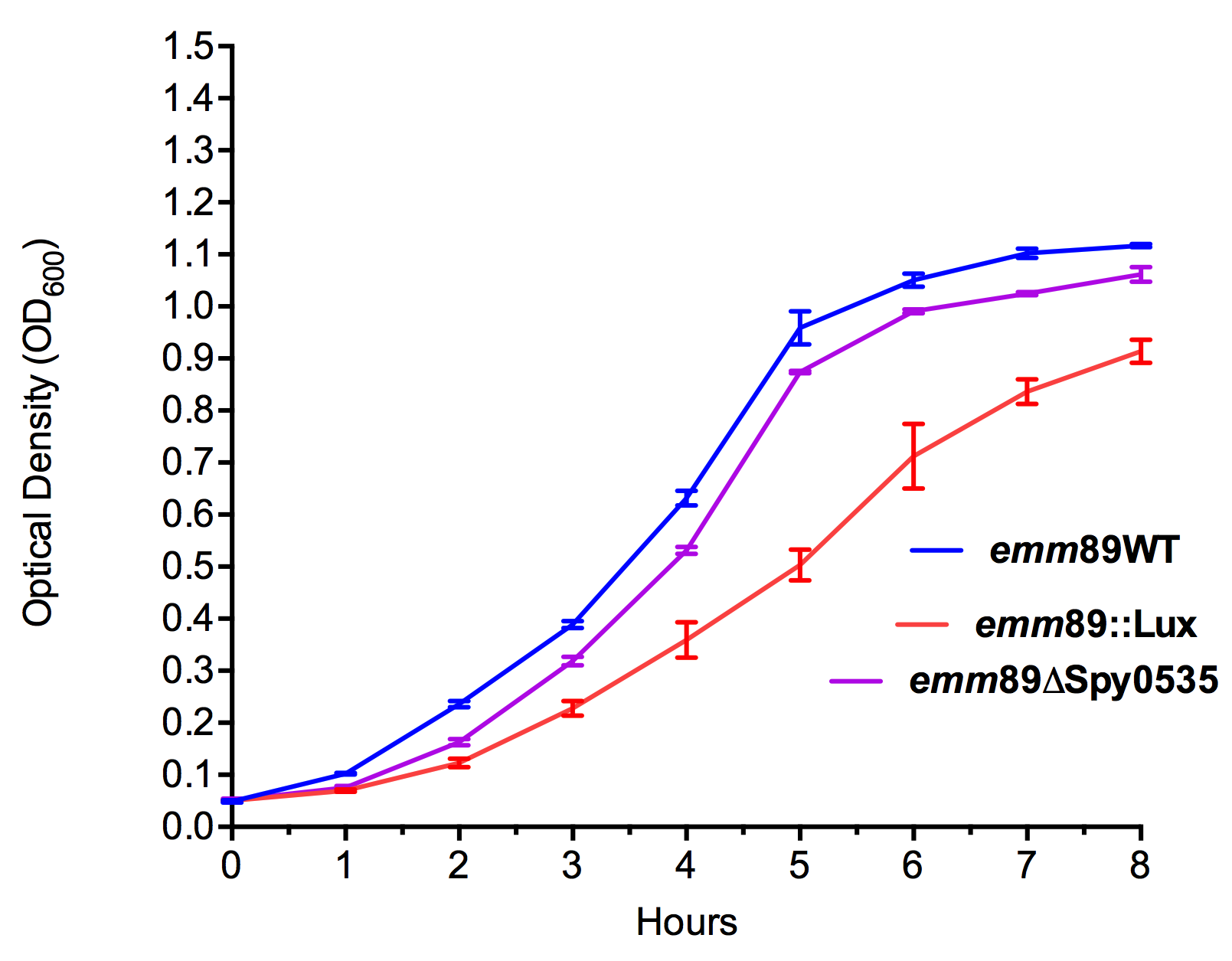

Supplement: Supplementary file 2 — Figure S2. Disruption of Spy0535 with plasmid control. Growth of M89 (blue), M89Δ0535 (purple, with disruption of Spy0535 using pUCMUTΔ0535) and M89::lux (red) was compared over 8 h. Mean and standard deviation of 3 biological replicate cultures are shown. (PNG 160 kb) [file 12866_2018_1200_MOESM2_ESM.png]

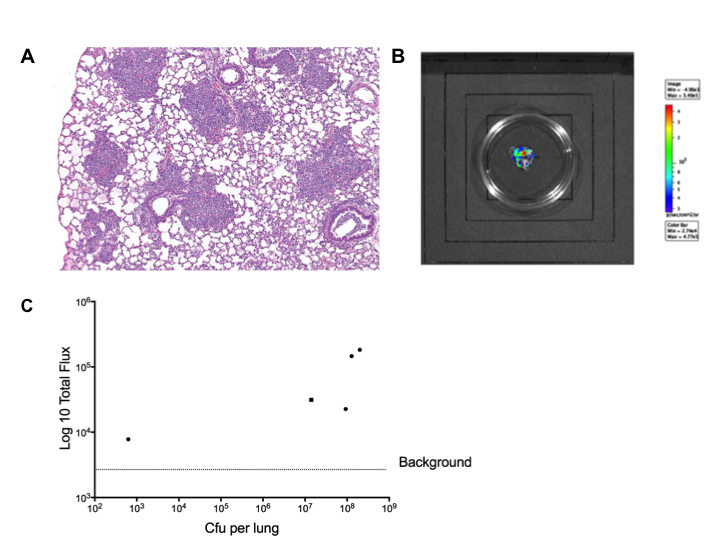

Supplement: Supplementary file 3 — Figure S3. Use of M89::Lux to model LRTI. A. Histopathology of lung tissue illustrating the inflammatory response 24 h following intranasal infection with M89::Lux. B. Light emitted from dissected mouse lung following direct intrathoracic injection of M89::Lux at 0 h; no light was visible through body wall. C Relation between light (total flux) and bacterial load (cfu) in dissected lung, n = 5. (PNG 307 kb) [file 12866_2018_1200_MOESM3_ESM.png]

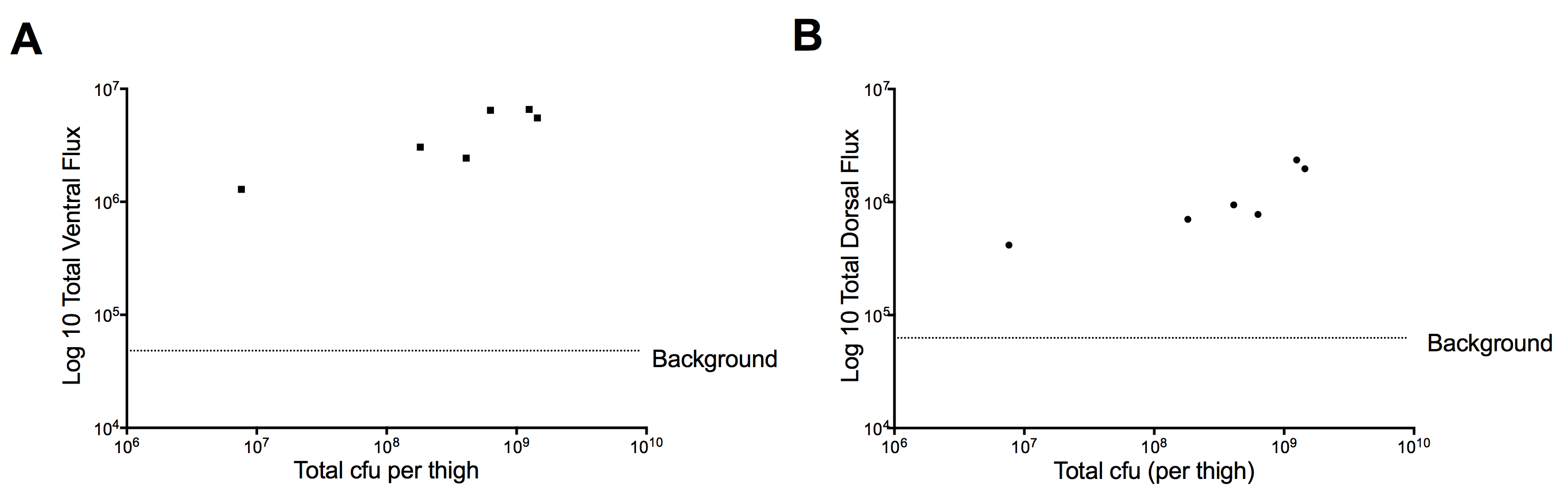

Supplement: Supplementary file 4 — Figure S4. Bacterial load and light emission following intramuscular infection with M89::Lux. A. Correlation immediately after infection between bacterial load in thigh muscle and total flux obtained dorsally using six mice, r2 = 0.88, (p < 0.05). B Lack of significant correlation between bacterial burden and total flux obtained ventrally (r2 = 0.77). (PNG 113 kb) [file 12866_2018_1200_MOESM4_ESM.png]

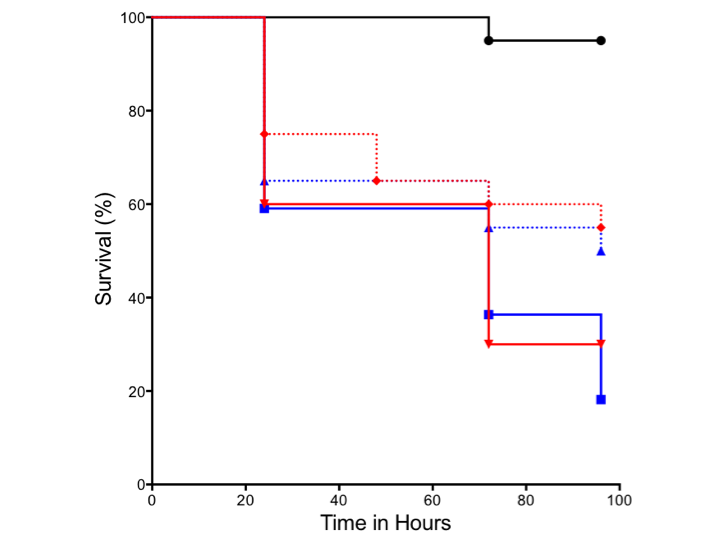

Supplement: Supplementary file 5 — Figure S5. Fitness burden conferred by bioluminescence in Galleria infection. Virulence of parent strains, M89 (blue line) and M75 (red line) was compared with bioluminescent derivatives M89::Lux (dotted blue line) and M75::Lux (dotted red line) in Galleria mellonella survival assays (n = 20 larvae per group). Larvae were inoculated with 1-2 × 10 6 cfu and monitored daily for survival over 5 days. Kaplan-Meier survival plots of infected larvae are shown compared with a PBS-inoculated group (controls – black line). Differences between strains were not significant. (PNG 38 kb) [file 12866_2018_1200_MOESM5_ESM.png]
